# Comparison of Hydrogels Based on Commercial Chitosan and Beetosan^®^ Containing Nanosilver

**DOI:** 10.3390/molecules22010061

**Published:** 2016-12-31

**Authors:** Bożena Tyliszczak, Anna Drabczyk, Sonia Kudłacik

**Affiliations:** 1 Department of Chemistry and Technology of Polymers, Cracow University of Technology, Warszawska 24, 31-155 Cracow, Poland; btyliszczak@chemia.pk.edu.pl; 2 Institute of Inorganic Chemistry and Technology, Cracow University of Technology, Warszawska 24, 31-155 Cracow, Poland; soniakudlacik@interia.pl

**Keywords:** chitosan, Beetosan^®^, silver nanoparticles, cell lines studies, swelling studies

## Abstract

Two series of hydrogels on the basis of commercial chitosan and chitosan derived from naturally expired honeybees are presented in this article. Sorption capacity and behavior of both kind of materials in simulated body fluids such as Ringer’s liquid or artificial saliva have been determined and compared. Presence of functional groups in synthesized materials have been determined by means of FT-IR spectroscopy. Structure and homogeneity of their surface have been defined using Scanning Electron Microscopy. Based on the conducted research, it can be stated that both chitosan and Beetosan^®^ hydrogels have very similar characteristics. It is worth noting that synthesis of such materials is environmentally friendly and leads to obtaining polymers that can be used for biomedical applications. Tested materials are characterized by low sorption capacity and do not have a negative impact on simulated body fluids. Moreover, based on the cell lines studies, it can be stated that Beetosan^®^ hydrogels have a negative influence on cells of cancerous origin and, what is important, significantly less adverse effects on fibroblasts.

## 1. Introduction

Nowadays, polysaccharides such as chitin, chitosan, cellulose, or hyaluronic acid are becoming more and more popular in applications in many realms including medicine, pharmacy, and even environmental protection. Particularly noteworthy is chitosan. Its growing interest stems from contemporarily desired features like non-toxicity, biodegradability, and biocompatibility. It is worth emphasizing especially the role that chitosan begins to play in controlled drug delivery systems. Rajan et al. [1] present an application of described polysaccharide as functionalized nanocomposites used in anticancer therapy. Such composites were encapsulated by cisplatin and it was stated that it is a promising nano-carrier. What is more, microparticles on the basis of chitosan in combination with gelatin contain a good solution for buccal delivery of propranolol hydrochloride as reported by Abruzzo et al. [2]. Bigucci et al. [3] propose an application of film prepared from chitosan and hyaluronan as a system for transdermal administration of thiocolchicoside, i.e., a medicament of anti-inflammatory and analgesic activity. This solution provides an alternative for the oral administration of this drug causing the extension of its effects. Chitosan also plays an important role in colon delivery of medicine used in case of bacterial infections such us vancomycin [4]. Apart from application of the mentioned biopolymer in delivery of pharmaceuticals, it is applied as a component of modern wound dressings [5,6,7,8]. Additionally, use a chitosan for preparation of hydrogels applied as sensors also seems interesting. Ebrahimi and Schonherr [9] describe a system composed of a chitosan-based hydrogel enriched with an appropriate substrate that could be used for detection of enzymes. 

The most common sources of chitosan are crustaceans such as shrimps or crabs containing this polysaccharide as the major component of their external shell. However, due to the increasing demand for this biopolymer in many fields, alternative sources are being considered. Insects such as crickets or bees represent an interesting source of chitosan. It should be noted that, in order to derive a desirable compound from these animals, a multistage chemical treatment is necessary. Substances such as waxes, mineral salts, proteins, or pigments should be removed from naturally expired honeybees in order to receive chitosan [10,11]. The process of deacetylation of attained chitin is also necessary and the resulting product, called Beetosan^®^, has a structure and properties similar to chitosan derived from standard sources such as prawns. 

Both chitosan and Beetosan^®^ contain a material which can be used for the preparation of hydrogels for biomedical applications. It follows the fact that they both are characterized by non-toxicity, biodegradability, and biocompatibility. Moreover, substances of different origin can be introduced into the matrix of hydrogels in order to give them a new property. Such an additive may be silver nanoparticles (AgNPs). They are well-known agents widely applied in medicine and pharmacy. Particularly noteworthy is an application of these kind of nanoparticles in antitumor therapy [12]. Silver nanoparticles are also characterized by biocidal activity against microorganisms such as viruses or bacteria. Due to this feature, they are applied as a component of modern wound dressings [13,14]. However, it should be mentioned that Storm-Versloot et al. [15] reported that any conclusive evidence of the impact of silver nanoparticles contained in the dressings on the prevention of wound infection as well as on stimulating the process of wound healing was not observed. Furthermore, it has been shown that the use of a silver compound, i.e., silver sulfadiazine (SSD), may result in a slower healing process. It was stated that a dose of applied silver compounds is important and after using too much of this substance undesirable effects can be observed.

Antimicrobial activity of silver nanoparticles results in their application in agriculture as an additive to agrochemical formulations or in food industry in the production of food packaging [16,17]. It is also worth mentioning that Greulich et al. [18] provided the information about the toxicity of silver nanoparticles on the cell of different origin (including human and bacterial). It therefore appears that the toxicity of the mentioned nanoparticles is not confined to the cells of pathogens. What is important in the case of both types of cells is that a toxic effect occurs by using a similar concentration of the antimicrobial medium. Hence, application of this additive in the form of an agent with antibacterial effect requires a series of detailed studies. 

## 2. Results and Discussion

### 2.1. Swelling Studies

**Figure 1:**
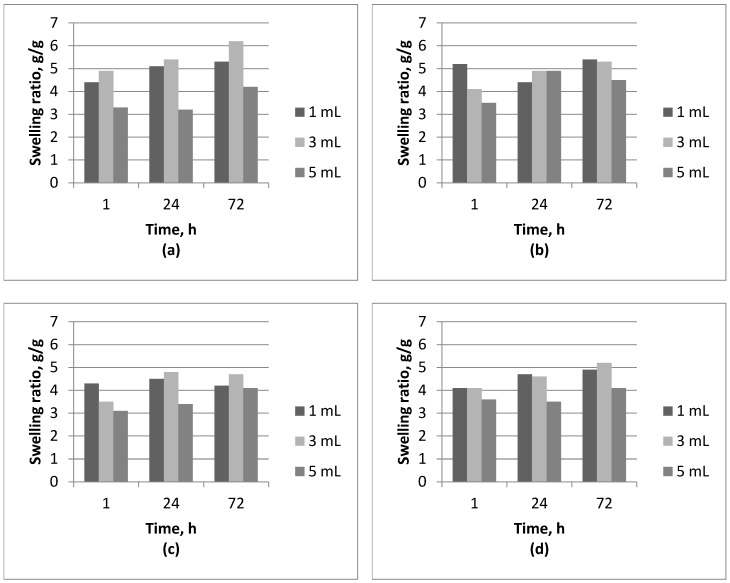
Comparison of swelling ability of Beetosan^®^ hydrogels in: (**a**) SBF; (**b**) artificial saliva; (**c**) Ringer’s liquid; and (**d**) distilled water.

**Figure 2:**
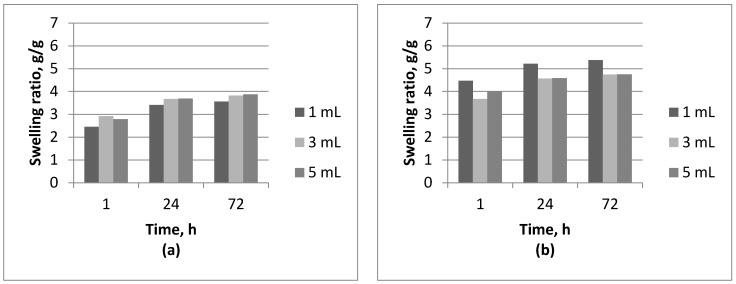

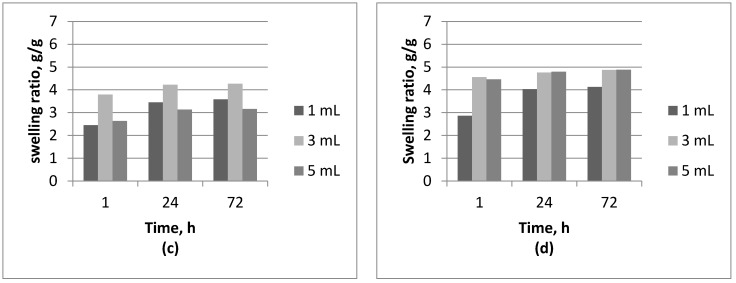
Comparison of swelling ability of chitosan hydrogels in: (**a**) SBF; (**b**) artificial saliva; (**c**) Ringer’s liquid; and (**d**) distilled water.By comparison, the study effects of the Beetosan^®^ materials with those obtained on the basis of commercial chitosan some similarities regarding the time and the liquid medium used are observed, however some differences between them also are visible (Figure 2). The addition of 5 mL of nanosilver to the hydrogels based on commercial chitosan results, in most cases, in an improvement of the sorption capacity. The only exception is observed in the case of Ringer’s liquid (Figure 2c). That can be due to an unfavorable arrangement of the hydrogel sample during the research, resulting in a smaller surface sorption.

Results of swelling studies are presented in Figure 1 and Figure 2. 

Based on the studies of swelling capacity of tested hydrogels it can be concluded that these materials exhibit the ability to swell. Their weight after immersion in aqueous solutions increases and is higher than the weight of the dry material (Figure 1 and Figure 2). Both the duration of the immersion and the medium in which the studies were conducted affect the swelling capacity of the tested materials. In case of hydrogels based on Beetosan^®^, the greatest ability to swell occurs within the first 24 h of immersion and has been maintained even for 72 h (Figure 1). The highest sorption capacity of these materials is observed in distilled water (Figure 1d), due to the fact that there are no other ions that can contribute to the increase of the crosslinking degree of the structure and that may reduce the swelling ability of the material. Swelling capacity also depends on the amount of nanoadditive introduced to the hydrogel matrix. It can be observed in each case that an introduction of nanoparticles in an amount of 5 mL to the hydrogel matrix causes a reduction of the sorption capacity (Figure 1). That may be caused by a retention of nanoparticles in the pores of the material, whereby the material's ability to absorb liquid is hampered. Reduction in the swelling ability of a given material may not necessarily be treated in negative terms. Sorption capacity can be regulated in a controlled manner and adjusted according to the individual needs of the application of such materials.

Based on the study it can be concluded that there is a difference in structure between commercial chitosan and the sort derived from bees. 

In chitosan derived from naturally expired honey bees—Beetosan^®^—a certain amount of chitin retained in the pores is present. Presence of this polysaccharide and silver nanoparticles introduced into the material cause a clogging of the pores. That results in reduction of swelling capacity of hydrogels obtained. 

### 2.2. Incubation Studies

**Figure 3:**
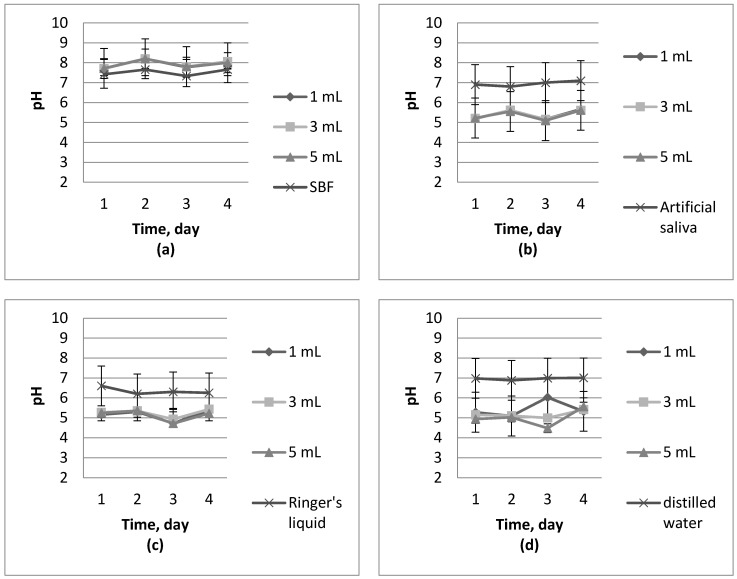
Incubation of Beetosan^®^ hydrogels in: (**a**) SBF; (**b**) artificial saliva; (**c**) Ringer’s liquid; and (**d**) distilled water.

**Figure 4:**
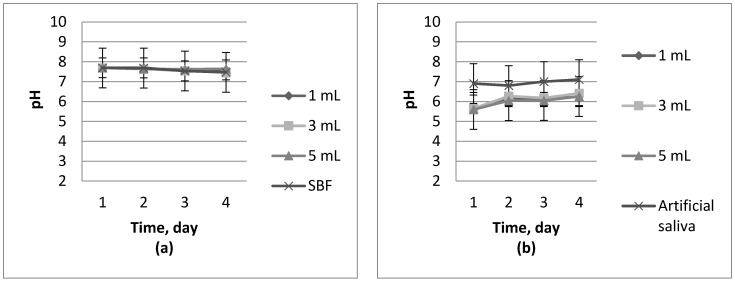

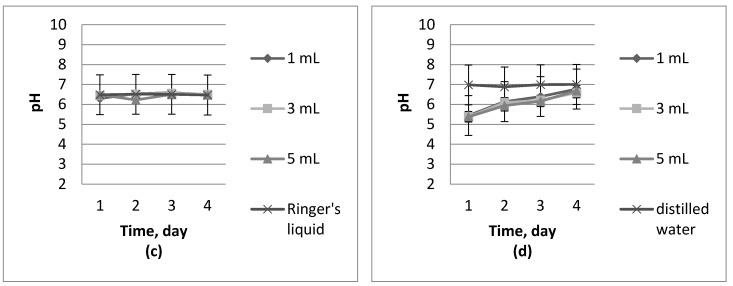
Incubation of chitosan hydrogels in: (**a**) SBF; (**b**) artificial saliva; (**c**) Ringer’s liquid; and (**d**) distilled water.

Results of pH measurements in separate liquids during incubation studies are shown in Figure 3 and Figure 4. 

Analyzing Figure 3 and Figure 4 during incubation experiments, no significant jumps or falls in pH values have been observed. Based on these observations, it can be concluded that the resulting material has not been degrading during four days of the research. Slow growth of pH at the beginning (for example Figure 4d) of the studies can be explained by the structural changes caused by a transition from a dry to a swollen state. On the basis of the above-listed graphs, it can be stated that the synthesized materials have a pH similar to the solution wherein incubation takes place. Hydrogels obtained are characterized by their buffering properties. These polymers are trying to maintain the pH values of a solution in which they are immersed at a constant level, slowly striving to achieve a pH level near 7. 

On the basis of conducted incubation studies, it can be concluded that the addition of nanosilver does not significantly affect changes in the pH of the solutions. What is more, any dependence of the change in pH values of the various solutions on the amount of nanosilver in the polymer matrix has not been observed. In some cases, the addition of silver nanoparticles slightly reduced pH value but that value was not dependent on the amount of additive introduced. pH changes, which are caused by the introduction of the additive to the hydrogel, oscillate around a value which can be acceptable since some of the fluids in the human body are characterized exactly by the same value. Conducted studies also confirm the similarity between chitosan derived from naturally expired honeybees—Beetosan^®^—and commercial chitosan.

### 2.3. Results of FT-IR Spectroscopy

In order to compare the molecular structure of hydrogels obtained on the basis of commercial chitosan and Beetosan^®^, FT-IR spectroscopy has been applied. Additionally, structures of both compounds constituting hydrogel matrixes have also been compared.

**Figure 5:**
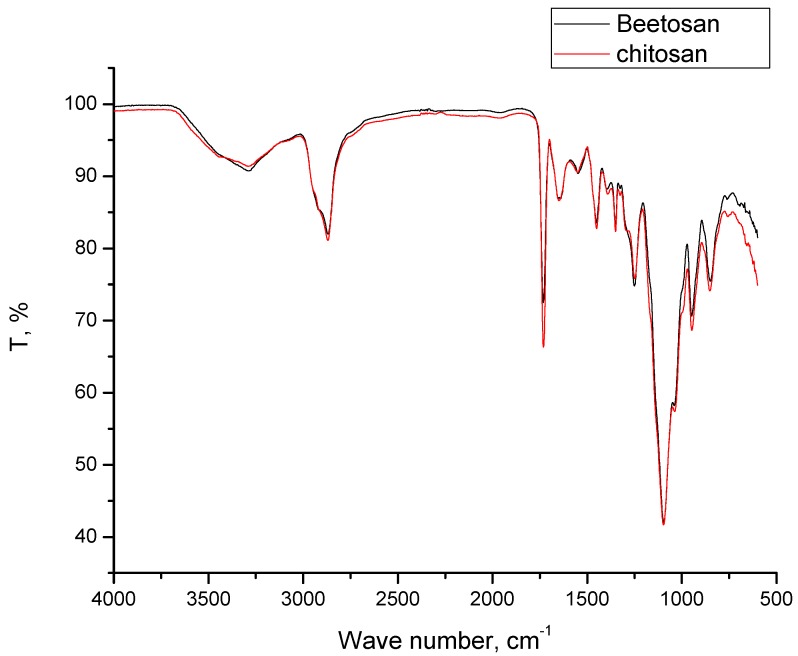
FT-IR spectra of hydrogels.

On the basis of the results shown in Figure 5, it can be stated that chemical structure of hydrogel based on chitosan derived from naturally expired honeybees—Beetosan^®^—is very similar to the structure of material on the basis of commercial one. Vibrations characteristic of the same functional groups were observed in both samples. Proposed chemical treatment of the abovementioned insects results in a material of the desired structure that is almost identical with the commercial product. That has been confirmed by FT-IR spectra derived from hydrogels based on these polysaccharides of a different origin. 

**Figure 6:**
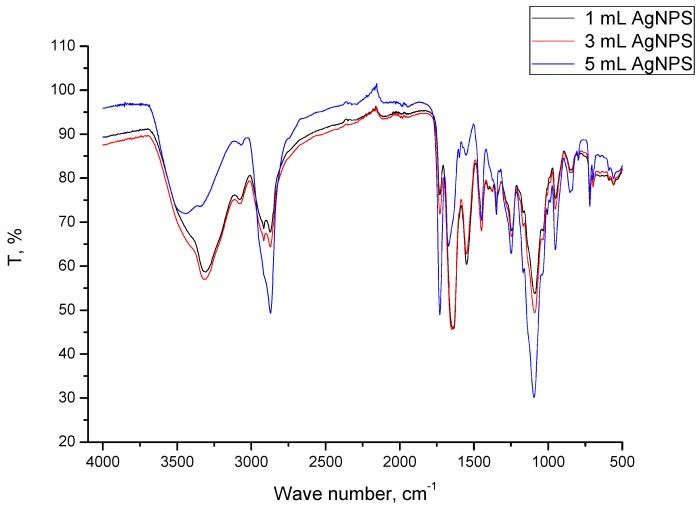
FT-IR spectra of Beetosan^®^ hydrogels modified with nanoparticles.

In Figure 6, the FT-IR spectra of Beetosan^®^ hydrogels modified with silver nanoparticles are presented. All observed vibrations are summarized in Table 1. It can be concluded that addition of nanoparticles into the hydrogel matrix has no significant effect on the structure of synthesized materials. The same vibrations were observed in unmodified hydrogels and also in materials with additives in their structure. 

### 2.4. SEM Analysis of Hydrogels

**Figure 7:**
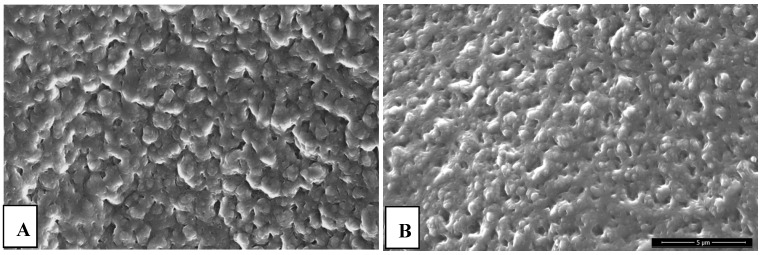
SEM microphotographs of chitosan hydrogels: (**A**) without additive; (**B**) modified with 5 mL silver nanoparticles.

**Figure 8:**
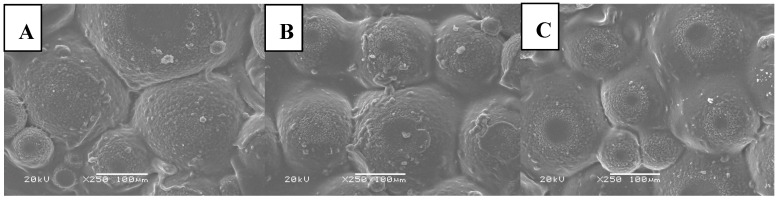
SEM microphotographs of Beetosan^®^ hydrogels modified with: (**A**) 1 mL; (**B**) 3 mL; (**C**) 5 mL of nanosilver.

Surface morphology of tested hydrogels is presented in Figure 7 and Figure 8.

SEM micrographs demonstrate heterogeneity of the surface of both kind of hydrogels. The materials obtained are characterized by porosity derived from the presence of free spaces between polymer chains in the structure of the hydrogels. Such structure can be compared to the three-dimensional network with a lot of crosslinked polymer chains and free areas in between filled with a liquid during absorption. However, it can be stated that addition of nanoparticles results in a slight decrease of the mentioned property, i.e., porosity. 

### 2.5. Results of Studies on Cell Lines

Results of cell lines studies are presented in Table 2Table 2. 

According to the cell lines studies, it can be concluded that synthesized samples based on Beetosan^®^ have a negative impact on Jurkat cells (of cancer origin) which may be deduced from the number of dead cells determined after first three days of the research. Of the analyzed cancer cells, 74% died after treatment with Beetosan^®^ hydrogel. It is worth noting that the negative effect on the cells was also observed after five and seven days of the research, after which, the next analyses of cell viability were conducted. On the other hand, 78% of fibroblasts remained alive after exposure to the same sample. 

## 3. Materials and Methods

### 3.1. Materials

Chitosan, 2-hydroxy-2-methylpropiophenon (used as a photoinitiator) and poly(ethylene glycol) diacrylate with average molecular weight Mn equal 700 g/mol (PEGDA 700, crosslinking agent) were obtained from Sigma Aldrich (Poznan, Poland). Gelatin was acquired from POCH SA (Gliwice, Poland). Chemicals used in studies were analytically pure. Beetosan^®^ was prepared at the Cracow University Technology (Cracow, Poland).

### 3.2. Methods

#### 3.2.1. Preparation of Beetosan^®^

Beetosan^®^ was obtained by multistage chemical treatment of naturally expired honeybees—the process carried out at Cracow University of Technology. Synthesis included removal of waxes, mineral salts, proteins, and pigments from the bees’ bodies. Then, material obtained was subjected to deacylation in order to produce Beetosan^®^.

#### 3.2.2. Preparation of Silver Nanoparticles

Silver nanoparticles were obtained by means of reduction of silver nitrate. Sodium citrate served as a reducing agent. 

#### 3.2.3. Preparation of Hydrogels

Preparation of hydrogel polymers requires a few steps. At first, required amounts of chitosan or Beetosan^®^ and gelatin are dissolved in acetic acid solution. Then, an appropriate amount of silver nanoparticle solution (at a concentration of 250 ppm) is added to the mixture. Next, crosslinking agent (PEGDA 700) and photoinitiator (2-hydroxy-2-methylpropiophenon) are introduced and the whole mixture is treated with UV radiation for 1–2 min. As a UV radiation source a quartz lamp—Emita VP-60 was applied. The appliance (Lodz, Poland) was characterized by the power of 120 W, used wavelength λ = 320 nm. Compositions of hydrogels are presented in Table 3. 

#### 3.2.4. Measurements of Sorption Capacity

Characteristic feature of hydrogels is their ability to reversible absorption of large quantities of liquids. In order to check the swelling ability of the prepared hydrogel samples, a weight of approximately 0.5 g was immersed respectively in 50 mL of distilled water, simulated body fluid (SBF, pH = 7.40), artificial saliva and Ringer’s liquid (solution isotonic with the human blood, containing the following ions (at a concentration given in mmol/L): Na^+^ = 147.2; K^+^ = 4.0; Ca^2+^ = 2.2; Cl^−^ = 155.7) for 1, 24 and 72 h. After each immersion, the hydrogels were separated from the solution, weighed, and immersed again. Swelling ability was determined by means of swelling ratio Q (g/g), which was calculated on the basis of the following equation:

$$Q=\frac{w-w_{0}}{w_{0}},$$

where w—weight of swollen sample (g), w_0_—weight of dry sample (g).

#### 3.2.5. Incubation Studies

Due to the potential use of prepared hydrogels for biomedical purposes, their behavior in relation to the simulated body fluid was determined. For this purpose, synthesized hydrogels were separately introduced into 50 mL of distilled water, Ringer’s liquid, and simulated body fluid (SBF) for 21 days. During the study, pH values of the particular solutions were monitored by carrying out measurement every two days. Incubation was carried out at 37 °C.

#### 3.2.6. FT-IR Spectroscopy

Hydrogels have been tested by application of Fourier transform infrared spectroscopy (FT-IR) with the use Spectrum 65 spectrometer (Perkin Elmer, Cracow, Poland). FTIR—ATR spectra were recorded at a temperature of 25 °C within the infrared range of 500–4000 cm^−1^. The ATR unit contained a diamond/ZnSe crystal. 

#### 3.2.7. SEM Analysis

Scanning electron microscopy (SEM) was used to define surfaces of attained materials. For this purpose Helios NanoLab H50HP equipment (Warsaw, Poland) was used. Sputtering samples with carbon was conducted at a uniform rate. The carbon layer was about 15 nm.

#### 3.2.8. Studies on Cell Lines

In order to check the toxicity of tested materials a selected sample—hydrogel based on Beetosan^®^ without addition of silver nanoparticles—was subjected to the research with cell lines. Studies of toxicity included two types of cells. First, the cell culture of Jurkat cells (tumor cells derived from T-cell acute lymphoblastic leukemia) was conducted for seven days. Second, the type of cells that were subjected to the prepared material were fibroblasts (WEHI cell line derived from mouse). Cell viability was analyzed by means of the cytometric method. Previously tested cells have been marked using propidium iodide (a compound that penetrates into dead cells).

## 4. Conclusions

Based on the studies described above, it can be concluded that the materials obtained on the basis of Beetosan^®^ can be an alternative to hydrogels received using chitosan derived from shellfish. These materials exhibit very similar characteristics. Such polymers, due to their ability to swell and biocompatibility, can be applied in areas such as medicine, pharmacy, tissue engineering, and nanotechnology. 

Synthesis of such materials is classified as an ecological method due to the short time of reaction, a small space of reaction, and no need to use organic solvents that have a very negative impact on the environment. The use of naturally expired honeybees in the synthesis of hydrogel materials is a remarkable step in this area due to the possibility of utilizing waste materials, that can be preferably used for biomedical applications. Introduction of silver nanoparticles into the hydrogel matrix modifies its features such as swelling ability. Presence of these nanoadditives can result in clogging pores of the material what may result in the decrease of sorption capacity. Furthermore, introduction of the mentioned additive has an impact on the porosity of the hydrogel. However, no changes associated with a behavior of hydrogel samples in contact with the simulated body fluid have been observed. That means that modified hydrogel maintains its biocompatibility. What is more, based on conducted cell line studies, it can be found that hydrogels based on Beetosan^®^ have a negative impact on cells of cancer origin. 

## Figures and Tables

**Table 1:** Vibrations of groups presented in tested hydrogels.

Range of Vibrations (cm^−1^)	Functional Groups	Type of Vibrations
3200–3600	–NH	stretching
3200–3500	–OH	stretching
2850–3000	–CH	stretching
1500–1650	–NH	deformation
1370–1390	–CH	deformation
1080–1360	–CN	stretching
1000–1300	–CO	stretching
600–700	–CH	deformation

**Table 2:** Results of cell line studies.

Tested cells	Day 3			Day 5			Day 7		
	% Total *	Alive **	Dead ***	% Total	Alive	Dead	% Total	Alive	Dead
Control	94	96	3	83	89	9	80	99	1
Jurkat cells	79	22	74	63	27	68	67	32	67
WEHI cell lines	45	78	22	-	-	-	-	-	-

Day 3, 5, 7—day when cytometric analysis using propidium iodide was carried out; *—% of analyzed cells (selected as suitable for the tests); **—% of alive cells from tested population; ***—% of dead cells from tested population.

**Table 3:** Compositions of prepared hydrogels.

	Gelatin 2% (mL)	Chitosan 3% (mL)	Beetosan^®^ 3% (mL)	AgNPs (250 ppm) (mL)	Crosslinking Agent (mL)	Photoinitiator (mL)
1.	20	30	-	1	8	0.25
2.	20	30	-	3	8	0.25
3.	20	30	-	5	8	0.25
4.	20	-	30	1	8	0.25
5.	20	-	30	3	8	0.25
6.	20	-	30	5	8	0.25
